# Suppression of Ribosomal Function Triggers Innate Immune Signaling through Activation of the NLRP3 Inflammasome

**DOI:** 10.1371/journal.pone.0036044

**Published:** 2012-05-14

**Authors:** Meghan L. Vyleta, John Wong, Bruce E. Magun

**Affiliations:** Department of Cell and Developmental Biology, Oregon Health and Science University, Portland, Oregon, United States of America; University of California Merced, United States of America

## Abstract

Some inflammatory stimuli trigger activation of the NLRP3 inflammasome by inducing efflux of cellular potassium. Loss of cellular potassium is known to potently suppress protein synthesis, leading us to test whether the inhibition of protein synthesis itself serves as an activating signal for the NLRP3 inflammasome. Murine bone marrow-derived macrophages, either primed by LPS or unprimed, were exposed to a panel of inhibitors of ribosomal function: ricin, cycloheximide, puromycin, pactamycin, and anisomycin. Macrophages were also exposed to nigericin, ATP, monosodium urate (MSU), and poly I:C. Synthesis of pro-IL-ß and release of IL-1ß from cells in response to these agents was detected by immunoblotting and ELISA. Release of intracellular potassium was measured by mass spectrometry. Inhibition of translation by each of the tested translation inhibitors led to processing of IL-1ß, which was released from cells. Processing and release of IL-1ß was reduced or absent from cells deficient in NLRP3, ASC, or caspase-1, demonstrating the role of the NLRP3 inflammasome. Despite the inability of these inhibitors to trigger efflux of intracellular potassium, the addition of high extracellular potassium suppressed activation of the NLRP3 inflammasome. MSU and double-stranded RNA, which are known to activate the NLRP3 inflammasome, also substantially inhibited protein translation, supporting a close association between inhibition of translation and inflammasome activation. These data demonstrate that translational inhibition itself constitutes a heretofore-unrecognized mechanism underlying IL-1ß dependent inflammatory signaling and that other physical, chemical, or pathogen-associated agents that impair translation may lead to IL-1ß-dependent inflammation through activation of the NLRP3 inflammasome. For agents that inhibit translation through decreased cellular potassium, the application of high extracellular potassium restores protein translation and suppresses activation of the NLRP inflammasome. For agents that inhibit translation through mechanisms that do not involve loss of potassium, high extracellular potassium suppresses IL-1ß processing through a mechanism that remains undefined.

## Introduction

Aberrant interleukin-1ß (IL-1ß) signaling has been implicated in a variety of inflammatory diseases ranging from arthritis to diabetes, making the manipulation of the IL-1 pathway an attractive therapeutic option for a growing number of pathologies that stem from innate immune activation [1], [2]. Critical to the efficacy of the innate immune system is the proper detection of invading microbes and toxic substances by macrophages that express pattern recognition receptors (PRRs) in the cytosol and at the cell surface. The Nod-like receptor (NLR) family member, NLRP3, is a cytosolic PRR that is activated by a large array of pathogen- and danger-associated molecular patterns to stimulate IL-1ß processing by a multiprotein complex termed the inflammasome [3]. The NLRP3 inflammasome consists of NLRP3, caspase-1, and the adaptor protein, ASC [3], [4], [5], [6]. Bacterial pore-forming toxins, viruses, asbestos, ATP, double-stranded RNA, and uric acid crystals all stimulate IL-1ß processing via NLRP3 inflammasomes [6], [7], [8], [9]. Although the importance of the inflammasome in mediating the release of IL-1ß from cells is well recognized, the mechanism(s) by which disparate activators trigger inflammasome activation are incompletely understood.

In macrophages, proinflammatory signals are required to mediate the expression of mRNA from the IL-1ß gene, resulting in the accumulation of pro-IL-1ß protein. These initial, or priming, signals are mediated by Toll-like receptor ligands such as lipopolysaccharide (LPS), which direct the NF-kappaB-dependent expression of pro-IL-1ß [3], [10]. The proteolytic processing of pro-IL-1ß by caspase-1 and the subsequent release of IL-1ß from cells requires a second signal to stimulate the assembly of inflammasome complexes. Loss of intracellular potassium has emerged as a frequent correlate of NLRP3 inflammasome activation and has been proposed to constitute one such signal. The conclusion that decreased intracellular potassium acts as a second signal to trigger activation of the NLRP3 inflammasome was based initially on the observation that loss of potassium induced by nigericin, a potassium ionophore, or by ATP results in the robust release of IL-1ß from cells in an NLRP3-dependent manner [7], [11]. However, the mechanism by which loss of intracellular potassium is linked with activation of the NLRP3 inflammasome is unclear. The production of reactive oxygen species (ROS) as a result of mitochondrial dysfunction has also been proposed as an activator of the NLRP3 inflammasome [8], [11], [12], [13], although the validity of this conclusion has been questioned [14], [15], [16].

It has been shown that sufficient levels of potassium are required for elongation of the peptide chain on the ribosome *in vitro* [17], [18], [19], [20] and that reduced concentrations of intracellular potassium fail to support protein synthesis in mammalian cells [20], [21]. Recently, we reported that ricin toxin, a well-defined inhibitor of protein synthesis, leads to IL-1ß- and macrophage-dependent inflammation when delivered to the lungs of mice [22]. By examining ricin-mediated release of IL-1ß from primary bone marrow-derived macrophages (BMDM), we found that the release of IL-1ß from BMDM requires the known components of the NLRP3 inflammasome: NLRP3, caspase-1, and ASC [23]. This finding led us to test whether inhibition of protein translation *per se* could constitute a signal that is sufficient to activate the NLRP3 inflammasome.

Here we employed a panel of well characterized protein synthesis inhibitors and found that each inhibitor induced the release of IL-1ß from LPS-primed BMDM in a manner that was dependent on the NLRP3 inflammasome. In BMDM treated with inhibitors of protein synthesis, intracellular potassium concentrations remained constant during the time in which IL-1ß release was observed from these cells, suggesting that activation of the NLRP3 inflammasome did not result from loss of potassium. To address whether inhibition of protein synthesis is a common feature of NLRP3 inflammasome activation, we examined two clinically relevant inflammasome triggers, monosodium urate, which causes inflammatory gout, and poly I:C, which mimics the proinflammatory actions of viral dsRNA. We report that monosodium urate and poly I:C each suppressed protein synthesis at doses and times that correspond with the release of IL-1ß from BMDM. We treated BMDM with NLRP3 stimuli in the presence of proteasome inhibitors, and found that proteasome activity was required for the ability of translational inhibitors, including MSU and poly I:C, to induce IL-1ß secretion from cells.

Our results demonstrate that impaired ribosomal function may constitute a common mechanism of NLRP3 inflammasome activation through a process that may require proteasomal activity. These studies further suggest that decreased cellular potassium may trigger IL-1ß release by inhibiting protein translation.

## Results

### Activation of the NLRP3 Inflammasome by Inhibitors of Translation

**Figure 1:**
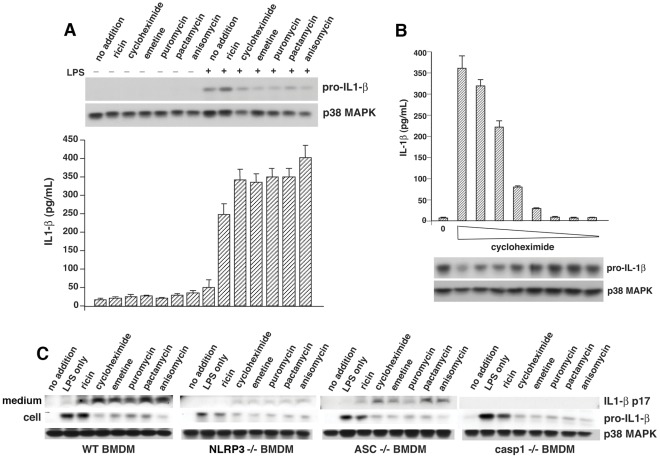
Inhibition of protein synthesis triggers secretion of IL-1ß from primed BMDM. A) WT BMDM were stimulated or not with 50 ng/ml LPS for 4 h followed by exposure to 10 µg/ml ricin, 25 µg/ml cycloheximide, 10 µg/ml emetine, 75 µg/ml puromycin, 0.2 µg/ml pactamycin, or 10 µg/ml anisomycin for 4 h prior to harvesting. Cell extracts were analyzed by immunoblotting for p38 MAPK (loading control) and pro-IL-1ß (upper panel). Media supernatants were analyzed by ELISA for released IL-1ß (lower panel). ELISA data are represented as mean ± SEM from triplicate dishes. B) LPS-primed WT BMDM were stimulated for 4 h with increasing doses of cycloheximide (from right to left: 0.03, 0.1, 0.3, 1.0, 3.0, 10, 30, and 100 µg/ml). Media supernatants were analyzed by ELISA for released IL-1ß (upper panel). Cell extracts were analyzed by immunoblotting for p38 MAPK (loading control) and pro-IL-1ß (lower panel). ELISA data are represented as mean ± SEM from triplicate dishes. C) Release of IL-1ß triggered by inhibitors of translation was determined by immunoblotting of cell extracts (cell) or culture medium (medium) from LPS-primed BMDM obtained from WT mice or mice deficient in NLRP3, ASC, or caspase-1, as indicated. P38 MAPK was loading control.

We tested the ability of a panel of well-characterized inhibitors of translation (cycloheximide, emetine, puromycin, pactamycin, and anisomycin) to stimulate IL-1ß release from BMDM via the NLRP3 inflammasome. When added at doses that suppress incorporation of [^3^H]-leucine by at least 90%, each inhibitor led to release of IL-1ß from LPS-primed BMDM by 4 h (Fig. 1A). Conversion of pro-IL-1ß to IL-1ß induced by cycloheximide proceeded in a dose-dependent manner (Fig. 1B), suggesting that the degree of translational impairment correlated well with the amount of IL-1ß that was released. As expected, there was a reciprocal relationship between the amount of pro-IL-1ß present in the cell lysate (Fig. 1A, upper panel) and the amount of IL-1ß detected in the culture medium by ELISA (Fig. 1A, lower panel). Cells remained strongly adherent to culture dishes at the termination of the experiments, and did not show morphological signs of apoptosis or activation of caspase-3 (not shown). To gain insight into the mechanism of IL-1ß release triggered by translational inhibitors, we exposed BMDM from mice deficient in NLRP3, ASC, or caspase-1 to agents that inhibited translation by a variety of mechanisms and compared their responses to those of wild-type (WT) cells. When BMDM deficient in NLRP3, ASC, or caspase-1 were exposed to the inhibitors, the cells secreted substantially reduced amounts of IL-1ß compared with WT BMDM exposed to the same inhibitors (Fig. 1C), suggesting that processing and release of IL-1ß were mediated through formation of the NLRP3 inflammasome.

### Extracellular Potassium Suppresses Release of IL-1ß Induced by Inhibitors of Translation

**Figure 2:**
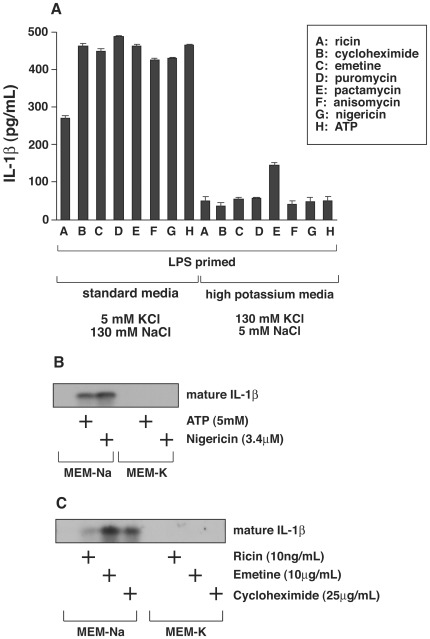
Effect of extracellular potassium on IL-1 processing and release. A) Bone marrow-derived macrophages were plated in triplicate wells in 12-well plates and primed with 50 ng/ml of LPS for 4 h. Cells were then incubated in medium containing 130 mM NaCl/5 mM KCl or 5 mM KCl/130 mM NaCl in the presence of absence of 0.01 µg/mL ricin, 25 µg/mL cycloheximide, 10 µg/mL, 10 µg/mL emetine, 75 µg/mL puromycin, 0.2 µg/mL pactamycin, 10 µg/mL anisomycin, 3.4 µM nigericin, or 5 mM ATP for 4 h. Medium was collected, and p17 IL-1 was determined by ELISA. B) Macrophages were plated, primed with LPS and incubated in medium containing 130 mM NaCl/5 mM KCl (MEM-Na) or 5 mM NaKCl/130 mM KCl (MEM-K) in the presence or absence 5 mM ATP or 3.4 µM nigericin for 4 h. Proteins were precipitated from the media with TCA and analyzed by Western blotting. C) Macrophages were plated, primed with LPS, and incubated in medium containing 130 mM NaCl/5 mM KCl (MEM-Na) or 5 mM NaKCl/130 mM KCl (MEM-K) in the presence or absence 10 ng/mL ricin, 10 µg/mL emetine, or 25 µg/mL cycloheximide for 4 h. Proteins were precipitated with TCA and analyzed by Western blotting.

Previous studies have described a requirement for potassium efflux by a variety of agents reported to activate the NLRP3 inflammasome, including nigericin and ATP [11]. Here, substitution of 130 mM Na^+^/5 mM K^+^ by 5 mM Na^+^/130 mM K^+^ in the culture medium suppressed the appearance of mature IL-1ß by translation inhibitors to a similar extent as nigericin and ATP, as detected by ELISA (Fig. 2A). Immunoblotting of the high potassium culture medium revealed undetectable amounts of mature IL-1ß in the culture medium of LPS-primed cells exposed to nigericin or ATP (Fig. 2B), or ricin, emetine, or cycloheximide (Fig. 2C).

### Lack of Correlation between Translation Inhibition and Potassium Efflux

The ability of high extracellular potassium to suppress activation of the NLRP3 inflammasome by multiple proinflammatory agonists [11] has been used to support a model demonstrating that decreased cellular potassium constitutes a general trigger for inflammasome activation. Initial studies were conceived because nigericin and ATP, agents that are known to cause potassium efflux [7], [24], are also potent activators of the NLRP3 inflammasome. The conclusion that loss of cellular potassium is responsible for activation of the NLRP3 inflammasome was based on the ability of high extracellular potassium to suppress pro-IL-1ß processing and release of mature IL-1ß in the presence of high potassium [11]. Subsequent studies have employed high extracellular potassium to block activation of the NLRP3 by a variety of agents [13], [25], [26], [27], [28], [29], [30]. Although these studies demonstrated that high extracellular potassium blocks release of IL-1ß, they did not determine whether the proinflammatory agents employed actually cause a loss of cellular potassium.

**Figure 3:**
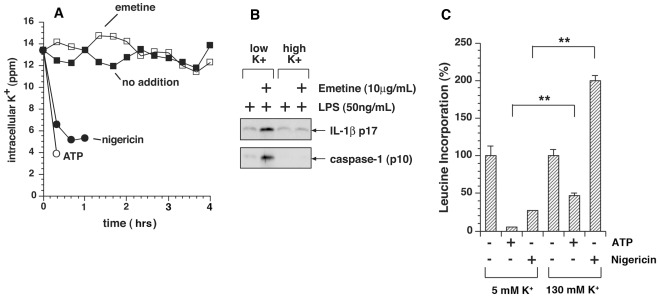
Inhibition of protein synthesis fails to elicit efflux of potassium from cells. A) Intracellular potassium in WT BMDM was analyzed by ICP-MS after priming with LPS for 4 h and exposure to emetine, ATP, and nigericin for indicated times. Data are represented as mean ± SEM from triplicate dishes. Measurements of potassium release by cells exposed to ATP and nigericin were terminated at 10 min and 60 min, respectively, as a result of cytotoxicity and detachment of cells from the dishes. Cells exposed to emetine showed no signs of cytotoxicity or detachment by 4 h. B) Elevated potassium blocks emetine-mediated release of IL-1ß and processing of caspase-1. LPS-primed BMDM were either exposed or not exposed to 10 µg/ml emetine for 4 h, at which time proteins were precipitated from the media with TCA and analyzed by Western blotting as shown. C) BMDM in triplicate wells were pulse-labeled in medium containing [^3^H]-leucine for 15 min prior to harvest at the indicated times and the amount of [^3^H]-leucine incorporation was measured.

To determine whether inhibition of translation would trigger efflux of potassium from macrophages, we exposed LPS-primed BMDM to emetine, a potent inhibitor of translation [31], [32] that irreversibly inhibits the elongation cycle of translation by greater than 99% within 1 minute [33] and produces release of IL-1ß within 4 h. Intracellular potassium was measured by inductively coupled plasma mass spectroscopy (ICP-MS). Nigericin and ATP, agents that have been shown to reduce intracellular potassium [7], [24], produced a 50% decrease in intracellular potassium concentration by 15 min and 45 min, respectively (Fig. 3A). BMDM exposed to nigericin and ATP detached from the culture dishes by 1 h. By contrast, cells exposed to emetine maintained normal levels of intracellular potassium for at least 4 h (Fig. 3A). Ricin and cycloheximide similarly failed to cause release of cellular potassium (not shown). These data suggest that in BMDM the inhibition of translation promoted the activation of the NLRP3 inflammasome but failed to induce leakage of cellular potassium in the interval of time (4 h) that preceded activation of the inflammasome and release of IL-1ß.

Four h after exposure of LPS-primed BMDM to emetine in medium containing low potassium, both IL-1ß and a proteolytic fragment of caspase-1 (p10) appeared in the medium (Fig. 3B, 2nd lane). In the presence of elevated potassium, neither p10 capase-1 nor IL-1ß appeared in the medium of cells exposed to emetine (Fig. 3B, 4th lane), suggesting that high potassium blocked caspase-1-mediated processing of pro-IL-1ß.

### Coupling between Potassium Efflux and Inhibition of Protein Translation

In view of the differential requirement for potassium efflux in promoting NLRP3 activation, we sought to determine whether nigericin, ATP, and panel of translation inhibitors that we employed shared any mechanistic features. It has been well established that the rate of protein synthesis is directly dependent on the concentration of intracellular potassium [18], [34] and that potassium ionophores inhibit translation at the same doses and kinetics as potassium efflux [35]. To test the ability of nigericin and ATP to suppress protein synthesis in BMDM, we subjected cells to nigericin or ATP and measured incorporation of [^3^H]-leucine. Exposure of BMDM to either nigericin or ATP triggered rapid inhibition of protein synthesis by 30 min in normal medium (130 mM Na^+^/5 mM K^+^; Fig. 3C). Replacement of extracellular Na^+^ by K^+^ (130 mM K^+^/5 mM Na^+^) partially reversed the blockade of protein synthesis in ATP-treated cells. Replacement of extracellular Na^+^ by K^+^ in nigericin-treated cells restored protein synthesis to a level even greater than that observed in untreated cells (Fig. 3C). These data are consistent with the known association of decreased cellular potassium with the inhibition of protein synthesis by nigericin, ATP, and other potassium ionophores [34], [35], [36].

### Monosodium Urate Crystals Suppress Protein Translation

**Figure 4:**
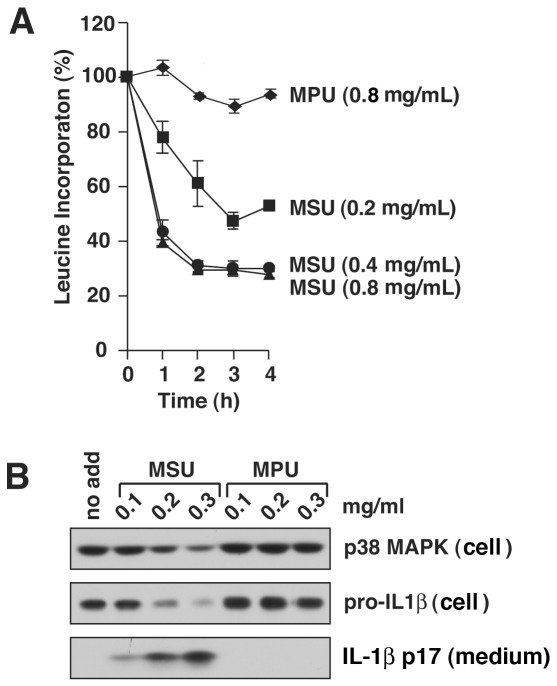
MSU crystals inhibit protein synthesis at concentrations that induce processing and release of IL-1ß. WT BMDM were primed with LPS for 4 h prior to exposure MSU or MPU at indicated concentrations. A) BMDM in triplicate wells were pulse-labeled in medium containing [^3^H]-leucine for 15 min prior to harvest at the indicated times and the amount of [^3^H]-leucine incorporation was measured. B) Cells were harvested 4 h after addition of indicated concentrations of MSU and MPU. Cell lysates (cell) and culture medium (medium) were examined by immunoblotting. P38 MAPK was loading control.

Gout is a chronic inflammatory response that is associated with the formation of crystals of monosodium urate (MSU). Incubation of LPS-primed murine macrophages with MSU crystals leads to the conversion and release of IL-1ß by a process that requires activation of the NLRP3 inflammasome. Because increased extracellular potassium blocks the activation of the NLRP3 inflammasome by MSU, it has been concluded that MSU acts through a mechanism that involves a decreased concentration of cellular potassium [11], [37], [38]. To determine if MSU inhibits protein synthesis at doses that stimulate release of IL-1ß from BMDM, we exposed cells to increasing doses of MSU or monopotassium urate (MPU), a salt of uric acid that is ineffective in promoting IL-1ß release [38]. The secretion of mature IL-1ß induced by MSU crystals was associated in a dose-dependent manner with inhibition of [^3^H]-leucine incorporation into BMDM (Fig. 4A,B). Monopotassium urate (MPU), which, as expected, failed to stimulate conversion of pro-IL-1ß to mature IL-1ß (Fig. 4B), also failed to inhibit protein synthesis as measured by incorporation of [^3^H]-leucine (Fig. 4A). These results demonstrated that MSU-induced suppression of protein synthesis in macrophages correlated with the release of IL-1ß by MSU.

### Double-stranded RNA Inhibits Protein Translation and Activates the NLRP3 Inflammasome

**Figure 5:**
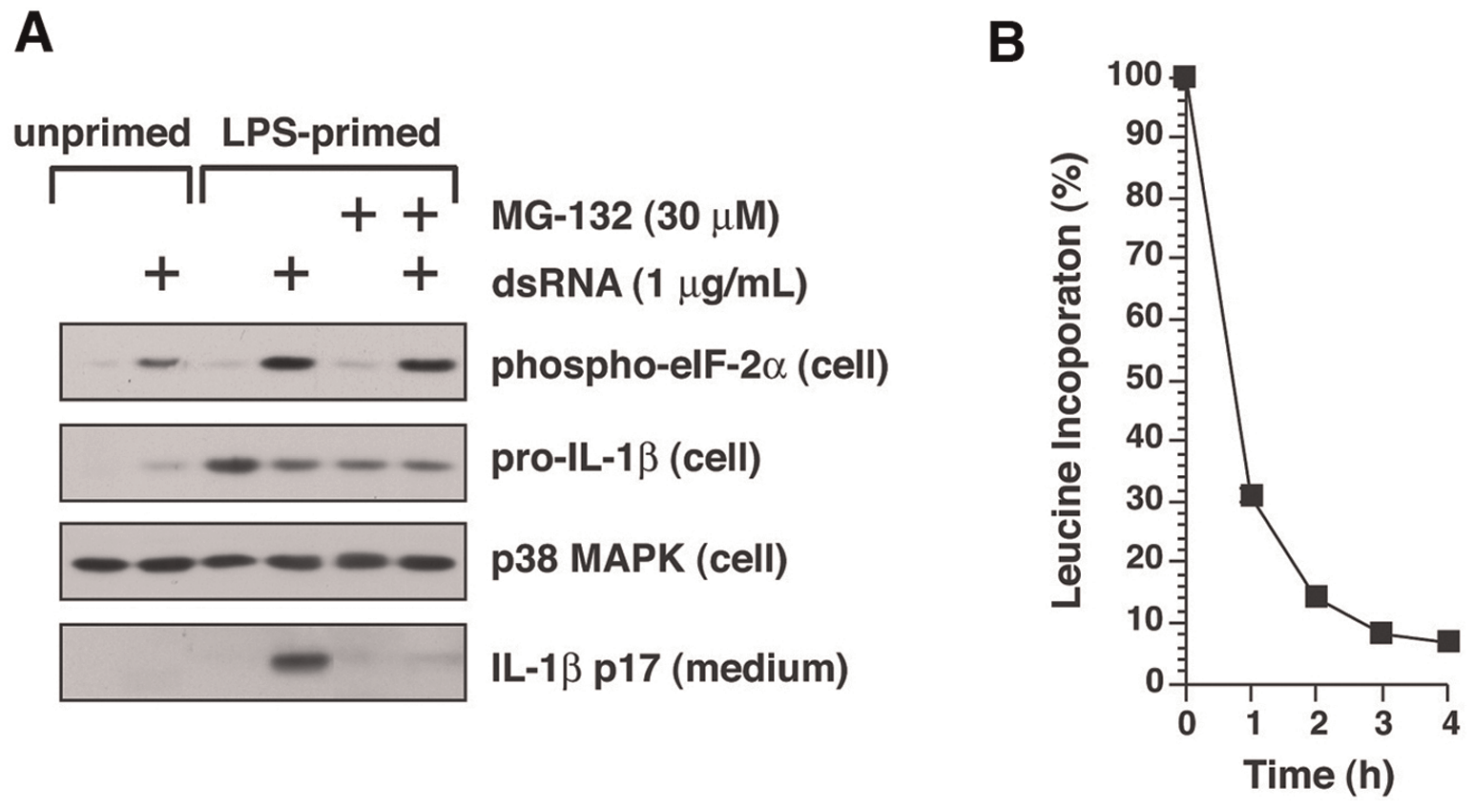
Inhibition of protein synthesis by dsRNA and inhibition of IL-1ß processing by MG-132. A) BMDM were treated with or without 4 h of LPS priming, as indicated. Cells were then rinsed in fresh medium and treated with either LipofectAMINE 2000 or LipofectAMINE 2000-poly I:C complex for 4 h, in the presence or absence of 30 µM MG-132, as indicated. Cell lysates (cell) or media (medium) samples were subjected to immunoblotting with the antibodies indicated. B) BMDM were treated with either LipofectAMINE 2000 alone or with LipofectAMINE 2000-dsRNA complex for the times indicated. Fifteen minutes before each time-point, 1 µCi of [^3^H]-leucine was added, and leucine incorporation was terminated by trichloroacetic acid. Each treatment was conducted in triplicate wells, and values are shown as mean ± S.D. Percent incorporation of [^3^H]-leucine at each point was calculated as the [^3^H]-leucine incorporated into cells exposed to LipofectAMINE 2000-dsRNA complex/[^3^H]-leucine incorporated into cells exposed to LipofectAMINE 2000 alone×100.

Many forms of cellular stresses, including exposure to dsRNA and accumulation of unfolded proteins [39], activate pathways that lead to the phosphorylation of the translation initiation factor eIF-2alpha on serine 51. Phosphorylation of eIF-2alpha on serine 51 results in global, frequently transient, inhibition of protein synthesis [39]. Intracellular poly I:C, an analog of dsRNA employed experimentally to mimic the effects of viral dsRNA in cells, has been shown to activate the NLRP3 inflammasome in BMDM [6]. Indeed, exposure of BMDM to poly I:C induced the release of IL-1ß from primed BMDM as well as the phosphorylation of eIF-2alpha on serine 51 (Fig. 5A). The release of IL-1ß from primed BMDM induced by poly I:C was accompanied by inhibition of protein synthesis (Fig. 5B).

### Proteasome Inhibitors Block Activation of the NLRP3 Inflammasome

**Figure 6:**
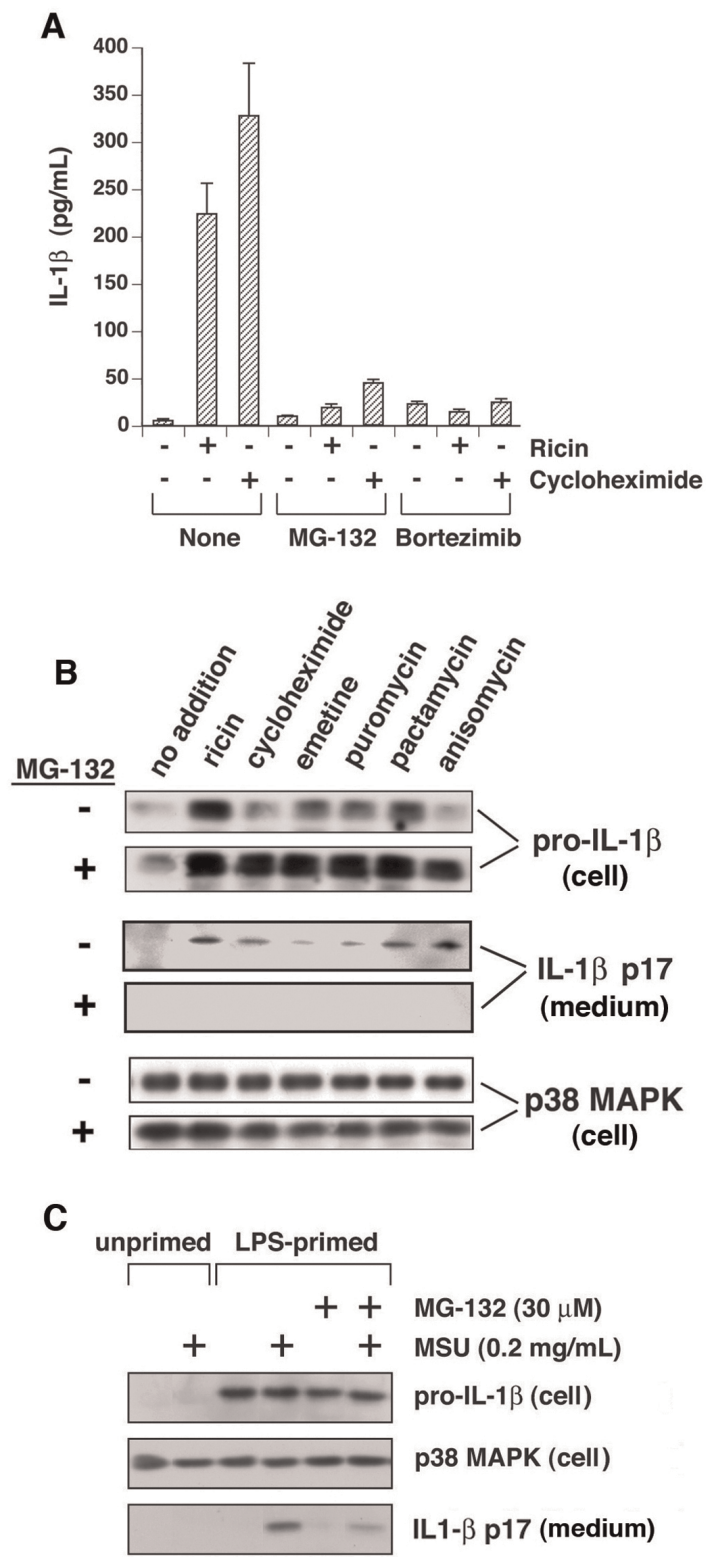
Proteasome inhibitors block processing and release of IL-1ß. A) LPS-primed WT BMDM were incubated in control medium or medium containing 10 ng/ml ricin or 25 µg/ml cycloheximide for 4 h. MG-132 (30 µM) or Bortezimib (0.5 µM) was included as indicated. Secreted IL-1ß was measured by ELISA in triplicate wells. B) LPS-primed WT BMDM were incubated in the presence or absence of MG-132 for 4 hours, in the presence or absence of inhibitors of protein synthesis, as indicated. Cell lysates (cell) and culture medium (medium) were examined by immunoblotting. C) LPS-primed or unprimed WT BMDM were or exposed to MSU, MG-132, or both for 4 h, as indicated. Cell lysates (cell) and culture medium (medium) were examined by immunoblotting.

Previously we showed that inhibitors of proteasome activity reduce the ricin-mediated activation of the NLRP3 inflammasome in LPS-primed BMDM [22]. Others have reported that proteasome inhibitors reduce the activity of the NLRP1 inflammasome but not the accumulation of pro-IL-1ß by anthrax lethal toxin [40]. To determine if proteasome inhibitors would block the ability of translation inhibitors to activate the NLRP3 inflammasome, we employed two proteasome inhibitors, MG-132 and bortezimib. IL-1ß release mediated by cycloheximide or ricin was strongly suppressed in LPS-primed BMDM co-treated with either bortezimib or MG-132 (Fig. 6A). Inclusion of MG-132 blocked the ability of each of the translation inhibitors to induce the processing of pro-IL-1ß and the release of IL-1ß from cells (Fig. 6B). MG-132 similarly suppressed the release of IL-1ß from cells exposed to MSU (Fig. 6C) and poly I:C (Fig. 5A), suggesting that proteasomes may participate more generally in activation of the NLRP3 inflammasome.

## Discussion

Ricin is a potent ribosomal toxin considered to be a potential bioterrorist agent due to the possibility of aerosol delivery to human populations [41]. Studies in animals demonstrated that delivery of ricin to the pulmonary system leads to acute lung injury and symptoms resembling acute respiratory distress syndrome [42], [43], [44], [45]. Previously we reported that the inflammatory and lethal effects of inhaled ricin were prevented in mice with a genetic deficiency in IL-1ß. Lung inflammation was blocked in mice that had been co-treated with IL-1 receptor antagonist (IL1RA; anakinra) or depleted of macrophages, suggesting the primacy of macrophage-derived IL-1ß in orchestrating pulmonary inflammatory responses to ricin [22]. Exposure of LPS-primed BMDM to ricin *in vitro* induced the processing pro-IL-1ß to mature IL-1ß in a NLRP3 dependent manner [23], raising the possibility that the toxic action of ricin on ribosomal function was responsible for activation of the NLRP3 inflammasome.

These results prompted us to examine the role that suppression of ribosomal activity plays in facilitating initiation of inflammatory signaling by IL-1ß. Indeed, the results presented here demonstrate that a panel of translation inhibitors, acting through different mechanisms on peptide initiation or elongation, promoted the conversion and release of IL-1ß by a process that required participation of the NLRP3 inflammasome (Fig. 1). These data led us to question whether inhibition of translation, which occurs as a result of a variety of cellular stresses, might serve as a relevant trigger for inflammasome activation in human inflammatory disease.

It has been proposed that decreased levels of intracellular potassium act to trigger release of IL-1ß as a result of activation of the NLRP3 inflammasome [11]. It is well documented that reduced concentrations of intracellular potassium also fail to support protein synthesis in mammalian cells [20], [21]. Potassium ionophores such as nigericin and valinomycin and compounds that poison the membrane-associated Na^+^/K^+^ ATPase rapidly trigger translational inhibition in cells concomitant with efflux of cellular potassium, and application of high extracellular potassium is sufficient to rescue ribosomal activity [21], [36], [46], [47]. Replacement of extracellular sodium with potassium suppresses the ability of nigericin and ATP to activate the NLRP3 inflammasome, supporting the notion that low intracellular potassium serves as a trigger for inflammasome activation [11]. Our results demonstrating the rescue of protein translation by increased extracellular potassium in BMDM exposed to nigericin or ATP (Fig. 3C) suggested to us that impairment of ribosomal activity may explain why loss of cellular potassium activates the NLRP3 inflammasome. However, the inability of emetine, a potent and irreversible inhibitor of protein synthesis, to promote efflux of potassium (Fig. 3A) demonstrated that an intracellular environment of low potassium was not required for activation of the NLRP3 inflammasome by molecules that directly interfere with ribosomal function.

It has been concluded that MSU crystals activate the NLRP3 inflammasome [48] through a process that leads to decreased concentration of cellular potassium [11], [38]. The conclusion that decreased concentration of cellular results from a leakage of potassium from cells after MSU treatment was based on the ability of high extracellular potassium (150 mM) to block processing of pro-IL-1ß to its active form [11]. Exposure of BMDM to MSU results in the engulfment of the insoluble crystals within the acidic milieu of endosomes [38], resulting in solubilization of the crystals and the subsequent rapid increase in cellular volume caused by release of sodium ions. The increase in cellular volume is thought to be responsible for a drop in concentration of intracellular potassium by dilution [38]. Our results showing that exposure of BMDM to MSU, but not MPU, results in the dose-dependent inhibition of protein translation and release of IL-1ß (Fig. 2), is consistent with the latter model and further supports the close association between inhibition of protein translation and activation of the NLRP3 inflammasome.

In view of our data showing that inhibition of translation fails to mediate loss of cellular potassium (Fig. 3), we were surprised that high extracellular potassium was able to block appearance of IL-1ß in the medium. Interestingly, Arlehamn et al. reported that high extracellular potassium inhibited IL-1ß release from cells after bacterial infection with *P. aeruginosa* and *S typhimurium*, which was dependent on the NLRC4 inflammasome, but that potassium leakage from cells could not be detected by flame photometry [49]. They conjectured that a minority of cells had undergone a loss of potassium due to the nature of the pathogens, which did not infect every cell, and for this reason they could not observe measurable potassium loss from the population of cells. Alternatively, high extracellular potassium may block the release of IL-1ß by a mechanism that is independent of intracellular potassium concentration. For example, high potassium (150 mM) has been shown to suppress activation and cleavage of recombinant caspase-1 *in vitro* [50]. Petrilli et al reported that MSU-treated primed macrophages release pro-IL-1ß and procaspase-1 into the medium, and that cells exposed to high extracellular potassium release pro-IL-ß and procaspase-1 into the high potassium medium, but that proteolytic processing of these proteins failed to occur [11]. These data and our data (Fig. 3B) suggest that high extracellular potassium may directly suppress the cleavage of pro-IL-1ß after externalization of the inflammasome complex by inhibiting the activation of procaspase-1. Our data suggest that cells in high potassium may suppress the activation of the NLRP3 inflammasome by two independent mechanisms: 1) by restoring intracellular potassium to normal levels in cells that have undergone leakage of potassium via pore formation (e.g. by nigericin) or stimulation of P2X7 receptors (e.g. by ATP), thereby preventing translational inhibition; and 2) by suppressing the activation of caspase-1 by an unknown mechanism.

The generation of reactive oxygen species (ROS) is commonly associated with NLRP3 inflammasome activation in response to a variety of agonists, including the mitochondrial inhibitors, rotenone and antimycin A [8], [11], [12], [13]. However, the conclusion that generation of ROS is responsible for inflammasome activation has been questioned [14], [15], [16]. Inhibition of mitochondrial Complex I or Complex III following exposure of cells to rotenone or antimycin A, respectively, leads to the generation of ROS through loss of mitochondrial membrane potential [51], [52], [53]. Rotenone and antimycin A have been shown to activate the NLRP3 inflammasome, presumably as a result of ROS production, since treatment of macrophages with Mito-Tempo, a scavenger of mitochondrial ROS, inhibited inflammasome activation [54]. Uncouplers of mitochondrial function such as rotenone and antimycin A are also potent inhibitors of translation, reducing the protein synthetic rate by more than 90% at concentrations employed to generate ROS in cultured cells [36], [55].

Several reports have demonstrated that ROS can inhibit mRNA translation [56], [57], [58]. Although the mechanism by which mitochondrial inhibitors inhibit protein synthesis is incompletely understood, it has been shown recently that mitochondrial inhibitors suppress protein synthesis by inducing the rapid phosphorylation of both eIF-2alpha and the elongation factor eEF2, presumably by stimulating PERK [59]. Indeed, peroxide- and hypoxia-mediated ROS have been shown to inhibit translation by increasing PERK- and PKR-mediated phosphorylation of eIF-2alpha and eEF2 [60]. Our data demonstrating that inhibition of translation can activate the NLRP3 inflammasome is consistent with the notion that the generation of ROS by mitochondrial dysfunction may activate the NLRP3 inflammasome by suppressing protein synthesis through stress-activated phosphorylation of eIF-2alpha and eEF2.

Inhibition of translation occurs in a variety of circumstances in nature, triggered by exposure to toxins, pathogens that co-opt host cell machinery, hypoxia, and sterile inflammatory signals released from damaged tissues. Phosphorylation of eIF-2alpha at Ser51 mediates translational inhibition in response to cellular signals [61], [62] by preventing the formation of the eIF2/GTP/Met-tRNA complex [63]. Stress-induced inhibition of translation through phosphorylation of eIF-2alpha is induced by viral dsRNA through activation of protein kinase R (PKR); hypoxia through the PRK-like endoplasmic reticulum kinase (PERK); and by glucose deprivation through activation of both PKR and PERK [39], [64], [65], [66]. An important consequence of eIF-2alpha phosphorylation is the regulation of gene expression, as mutations that interfere with eIF-2alpha phosphorylation lead to defective expression of stress-induced genes [67]. Recent evidence suggests that PKR acts as a central integrator in the inflammatory component of metabolic control by linking nutrient- and pathogen-sensing pathways in development of insulin resistance, type 2 diabetes, and other chronic metabolic pathologies [68]. Poly I:C-mediated activation of the NLRP3 inflammasome has been previously reported [6]. In LPS-primed BMDM we found that poly I:C mediates eIF-2alpha phosphorylation, inhibition of protein synthesis, and the release of IL-1ß (Figure 5). Phosphorylation of eIF-2alpha is required not only for attenuation of translation, but also for transcriptional induction and survival in response to endoplasmic reticulum-mediated stress (ER stress) [67]. ER stress activates the NLRP3 inflammasome via a pathway that does not involve the unfolded protein response [27]. Repression of translation through phosphorylation of eIF-2alpha leads to activation of NF-kappaB and the subsequent transcription of NF-kappaB-directed genes by promoting the turnover of the labile inhibitor, IkappaB alpha protein [69]. Stress-induced translational inhibition by phosphorylated eIF-2alpha may contribute to inflammatory responses by simultaneously promoting the two necessary events required to produce IL-1ß: the NF-kappaB-mediated synthesis of pro-IL-1ß and the release of IL-1ß through activation of the NLRP3 inflammasome. This model could explain how stress signals that converge on eIF-2alpha could induce IL-1ß-dependent inflammatory responses.

Maintenance of the intracellular level of proteins that exhibit short half-lives, such as p53 and IkappaB, is frequently regulated by the balance between their rate of synthesis and proteasome-directed degradation [70], [71], [72]. For example, inhibition of protein translation by stress-induced phosphorylation of eIF-2alpha leads to activation of NF-kappaB through proteasome-dependent degradation of IkappaB [69], [73]. Our experiments determined that co-treatment of BMDM with proteasome inhibitors plus ricin, cycloheximide, puromycin, pactamycin, anisomycin, MSU, or dsRNA led to a reduction or complete suppression of IL-1ß release as measured by ELISA (Figure 4A) or immunoblot (Figures 4B, 5). LPS-primed cells treated with poly I:C in the presence of MG-132 exhibited a reduction in IL-1ß release but did not change the phosphorylation status of eIF-2alpha (Fig. 5), suggesting that involvement of the proteasome in activating the inflammasome is positioned downstream of translational inhibition in these cells.

In light of our data showing a link between suppression of protein synthesis and activation of the NLPR3 inflammasome, we propose that labile protein(s) may suppress the formation of the NLRP3 inflammasome. In such a scenario, inhibition of translation, which accompanies many types of cellular stresses, would lead to a decrease in abundance of putative repressor protein(s), perhaps through proteasome-mediated degradation. In this model, blockade of proteasomal activity would extend the lifetime of the putative repressor protein(s)**.** The validity of this model would require identification of labile protein(s) that inhibit the processing of pro-IL-1ß.

**Figure 7:**
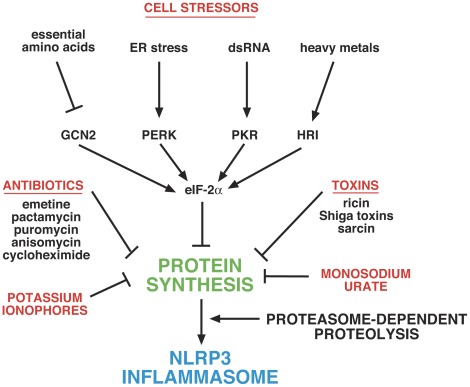
Graphic depiction of proposed mechanisms by which different stimuli activate the NLRP3 inflammasome. Inhibition of protein synthesis leads to activation of the NLRP3 inflammasome by preventing the synthesis of short-lived inhibitory protein(s) that are degraded by proteasomes. Inhibition of protein synthesis is mediated by a variety of physiological cell stressors acting through phosphorylation of eIF-2alpha, leading to a transient decrease in protein synthesis. Toxins such as ricin, Shiga toxins, and sarcin inhibit protein synthesis by interfering with the structure of the sarcin/ricin loop of 28S rRNA. Antibiotics act through various ribosome-associated mechanisms to inhibit translational initiation and/or elongation. Potassium ionophores (and receptors such as P2X7) inhibit translation by mediating loss of cellular potassium. Monosodium urate inhibits translation by inducing cell swelling, which leads to dilution of intracellular potassium.

The current study demonstrates that suppression of ribosomal function by molecules acting by disparate mechanisms is sufficient to activate the NLRP3 inflammasome. These data suggest that inhibition of translation may constitute a common stimulus by which seemingly dissimilar activators promote the processing and release of IL-1ß. A decreased rate of translation may constitute a regulatory node that integrates signals from toxins, pathogens, and metabolic disturbances, thereby enhancing systemic inflammation by promoting the processing and release of IL-1ß. The suppression of IL-1ß release by proteasome inhibitors suggests that labile protein(s) may be responsible for blocking the activation of the NLRP3 inflammasome under non-stressed conditions. A decrease in translation rate may lead to reduction in cellular levels of these protein(s), thereby leading to formation of active NLRP3 inflammasomes. Further studies that focus on identification of labile inhibitors of inflammasome function are clearly necessary to test the validity of this hypothesis. A graphic depiction of the proposed mechanisms underlying NLRP3 activation by different stimuli is shown in Figure 7.

## Materials and Methods

### Ethics Statement

This study was approved by the Institutional Animal Care & Use Committee (IACUC) and the Institutional Biosafety Committee of OHSU. The authors have conformed to the policies of the Institutional Office of Integrity of OHSU. Mice are anesthetized with ketamine/xylazine. To minimize pain and discomfort, mice are euthanized by cervical dislocation as per the recommendation of the Panel of Euthanasia of the American Veterinary Medical Association. Only personnel trained in animal experimentation are involved. All procedures that involve the use of ricin and animals have been approved by the Chemical Safety Officer at OHSU and by the Institutional Animal Care Utilization Committee (IACUC) at OHSU. Personnel are gloved and wear protective coverings over their body, including full face respirator masks. All animal studies are conducted in chemical safety hoods. Animals are housed in designated areas as approved by the IACUC under protocol A900, OHSU.

### Reagents and Antibodies

Cycloheximide, emetine, anisomycin, and puromycin were purchased from Sigma-Aldrich (St. Louis). Ricin was purchased from Vector Laboratories (Burlingame, CA). Polyinosinic-cytidylic acid (Poly I:C) was purchased from Midland Certified Reagents (Midland, Texas). Lipofectamine 2000 was purchased from Life Technologies (Carlsbad, CA). Bortezimib was purchased from LC Laboratories (Woburn, MA). MG-132 was purchased from EMD Biosciences (Gibbstown, NJ). Pactamycin was a gift from the Upjohn Company (Kalamazoo, MI). Anti-IL-1ß was purchased from Abcam (Cambridge, MA) and anti-p38 MAPK was purchased from Santa Cruz Biotechnology (Santa Cruz, CA). Anti-phospho-eIF-2alpha (ser51) #9721 was purchased from Cell Signaling (Danvers, MA). The mouse IL-1ß enzyme-linked immunosorbent assay (ELISA) Ready-Set-Go was purchased from eBioscience (San Diego, CA). Crystals of MSU and MPU were prepared as described [74].

### Animals and Animal Procedures

All animal procedures were performed according to protocols that have been approved by the Institutional Animal Care and Use Committee at Oregon Health and Science University, Portland, Oregon. C57BL/6J and caspase-1 deficient mice were purchased from the Jackson Laboratory (Bar Harbor, ME). ASC- and NLRP3-deficient mice were kindly provided by V. Dixit (Genentech, San Francisco, CA). Male mice, 8–10 weeks of age, were used throughout the experiments.

### Isolation of Bone Marrow- Derived Macrophages (BMDM)

Male mice, 8 to 10 weeks of age, were used throughout the experiments. Cells were prepared from WT C57BL/6J, ASC-, Caspase-1-, and NLRP3-deficent mice. Marrow was flushed from femurs and tibias with PBS and cultured in alpha-Minimum Essential Medium (αMEM, Cellgro, Herndon, VA), supplied with 10% Fetal Bovine Serum (FBS, Cellgro, Herndon, VA), 50 µg/ml gentamicin, and 100 ng/ml recombinant mouse Colony Stimulating Factor 1 (CSF-1, R&D Systems, Minneapolis, MN) for 72 hrs on non-tissue culture treated 10-cm Petri dishes. BMDM were passaged and cultured for an additional 72 h. Each confluent 10-cm dish was transferred into one 6-well or one 12-well tissue culture plate and cultured for 24 hrs before initiating experimental treatment.

### Treating BMDM

Cells were serum deprived in alpha-MEM for 30 minutes followed by treatment for 4 hours in the presence or absence of 50 ng/mL LPS. Cells were then rinsed and fresh media was added followed by exposure to the indicated doses of inhibitors (or solvent control), in the absence of LPS, for 4 hrs. In experiments employing elevated potassium, sodium was replaced by potassium at an equivalent molar concentration.

### Immunoblotting

BMDM cells were lysed in 2×ESB lysis buffer in preparation for immunoblotting. Equal volumes of the cell lysates were separated on a 10% denaturing polyacrylamide gel in the presence of sodium dodecyl sulfate and were transferred onto polyvinylidene difluoride membranes according to standard laboratory procedures. For detection of IL-1ß released from cells, proteins from media supernatants were precipitated using TCA plus 200 µg insulin carrier protein and separated on 13% gels. Membranes were incubated with the indicated antibodies and the corresponding horseradish peroxidase-conjugated secondary antibodies. Signals were detected by using enhanced chemiluminescence.

### ELISA

Media from BMDM were collected and analyzed in triplicate using IL-1ß ELISA (eBioscience) according to the manufacturer’s protocol.

### ICP-MS

Primed BMDM were exposed to inhibitors of protein synthesis for indicated times up to 4 h and cells were digested overnight in culture dishes in 1 mL 10% HNO_3_. Digests were analyzed by inductively coupled plasma mass spectrometry (ICP-MS) in the Metal Ion Core by Dr. Martina Ralle in the Department of Biochemistry and Molecular Biology at OHSU. ICP-MS analysis was performed using an Agilent 7700× system equipped with an ASX-250 Autosampler. The system was operated at a radio frequency power of 1,550 W, an argon flow rate of 15 L/min, carrier gas flow rate of 1.04 L/min, and helium (He) gas flow rate of 4.3 ml/min (only in He mode). Data were quantified using a seven-point (0, 1, 10, 100, 1,000, 2,000, and 5,000 ppb (ng/g)) calibration curve with an external standard for potassium. All data were acquired in He mode to remove interference from oxides, argides, and chlorides. For each sample, data were acquired in triplicate and averaged. Internal standards introduced with the sample were used to correct for plasma instabilities. A National Institute of Standards and Technology standard reference material (SRM 1577c) was used to ensure elemental recovery of >90%.

### Measurement of Protein Synthesis via Incorporation of [^3^H]-leucine

BMDM were cultured in 24-well tissue culture plates. Treatments were performed in leucine-free/serum-free Dulbecco modified Eagle medium, for the indicated times. Prior to harvesting, the cells were pulse-labeled with 1 µCi of [^3^H]-leucine in 1 ml of leucine-free DMEM for times specified in the figure legends. Ten percent trichloroacetic acid was added to terminate incorporation. Wells were washed in water and 88% formic acid was added to solubilize the trichloroacetic acid-insoluble proteins. The samples were counted in a liquid scintillation counter. In each experiment, triplicate wells were used per experimental point.

### Statistical Analysis

Individual groups were compared using unpaired *t* test analysis and were interpreted in a two-tailed manner.
